# The Impact of an Early Eclectic Rehabilitative Intervention on Symptoms in First Episode Depression among Employed People

**DOI:** 10.1155/2013/926562

**Published:** 2013-11-10

**Authors:** Tero Raiskila, Sanna Blanco Sequeiros, Jorma Kiuttu, Marja-Liisa Kauhanen, Kristian Läksy, Kirsi Vainiemi, Annamari Tuulio-Henriksson, Helinä Hakko, Matti Joukamaa, Juha Veijola

**Affiliations:** ^1^School of Health Sciences, University of Tampere, 33014 Tampere, Finland; ^2^Department of Psychiatry, University Hospital of Oulu, Institute of Clinical Medicine, University of Oulu, P.O. Box 5000, 90014 Oulu, Finland; ^3^Department of Psychiatry, University of Oulu, P.O. Box 5000, 90014 Oulu, Finland; ^4^Lapland Central Hospital, Department of Psychiatry, P.O. Box 8041, 96101 Rovaniemi, Finland; ^5^The Social Insurance Institution, Kela, P.O. Box 20, 00232 Helsinki, Finland; ^6^Oulu Deaconess Institute, Albertinkatu 16, 90101 Oulu, Finland; ^7^Department of Behavioral Sciences, University of Helsinki, 00014 Helsinki, Finland; ^8^Tampere University Hospital, Department of Psychiatry, Tampere, Finland

## Abstract

*Objective*. To evaluate the effect of an early vocational-orientated eclectic intervention on beck depression inventory (BDI) scores compared to treatment as usual in first ever depressive episode among employed people. *Design*. A randomized controlled trial comparing the rehabilitative intervention and the conventional treatment. *Subjects*. The subjects came from occupational health care units. *Methods*. Employees were sent to a rehabilitation center after being screened for depression using the BDI. They were diagnosed using the structured clinical interview for DSM-IV. The participating subjects (*N* = 283) were randomized into intervention and control groups. The intervention group received eclectic early depression intervention treatment (*N* = 134) and the control group was treated in the conventional way (*N* = 100). They were followed for one year. *Results*. The mean decrease in BDI scores within the intervention group was from 20.8 to 11.6 and within the control group from 19.3 to 10.8. BDI score decreased by 10 or more points in 64% of the participants in the intervention group and in 53% of the control group (*P* = 0.013). *Conclusions*. There was some evidence that early eclectic intervention in first ever episode depression may be more effective than conventional treatments among working age people in employment.

## 1. Introduction

Depression is a common psychiatric disorder characterized by high rates of relapse and recurrence [1]. Treatment results in depression are not always satisfactory. According to the STAR* D study, the overall cumulative remission rate when using antidepressive medication in the treatment of major depressive disorder (MDD), after four treatment steps, was less than 70% [2].

A review by Baumeister and Hutter [3] concluded that single interventions have little effect on outcomes in depressive patients. Instead, collaborative care interventions that focus on the work and family relations of an individual, and involve occupational health care workers and staff from psychiatric and psychological facilities, are efficacious in patients with depression. In contrast, a systematic review by Furlan et al. [4] concluded that there is insufficient evidence to determine which interventions are effective in managing depression in the workplace. 

Several studies have stressed the importance of psychiatric vocational rehabilitation programs, including supported employment models with high levels of integration of psychiatric and vocational services and different psychosocial interventions designed to prevent prolonged working disability [5–7]. The early eclectic rehabilitative intervention program (EERIP) is a relatively new practice used to help working age people with various levels of depression [8]. This intervention comprises a psychologically orientated vocational rehabilitation program, which addresses the specific needs of people in employment.

The aim of this study was to examine the effect of the EERIP on depressive symptoms in subjects with an acute presentation of first ever depression. We hypothesized that the intervention program would be effective in reducing depressive symptoms.

## 2. Materials and Methods

### 2.1. Design

**Figure 1:**
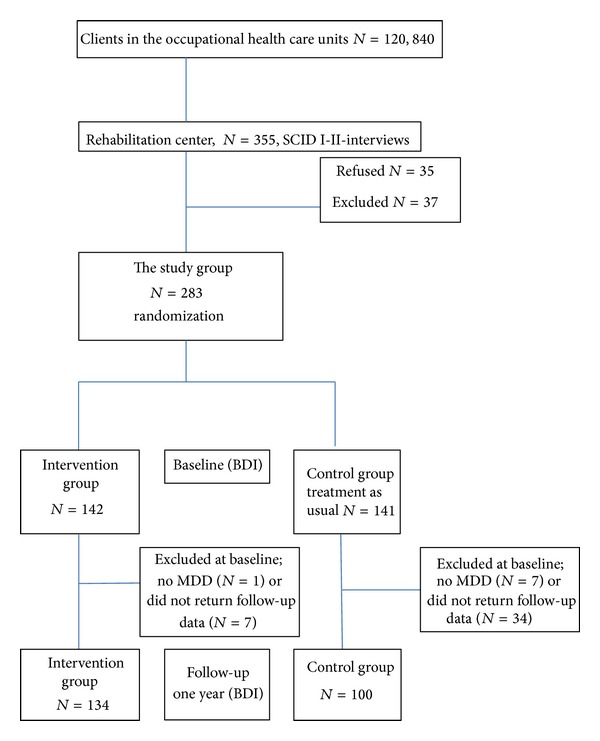
The flow chart of subjects randomized and followed by Beck Depression Inventory (BDI).

The present study forms part of a rehabilitation project, designed to measure the effectiveness of an EERIP on first ever depressive disorders among employed persons (18–64 years) in Finland. The study design, recruitment, and methods have been described in detail previously [9]. The participants were recruited from 18 occupational health care units in Northern Finland between the years 2004 and 2009 (Figure 1). Eligible subjects were randomized into interventional and control groups. A two-phase rehabilitation program was used for the intervention group; the control group received treatment as usual (TAU).

### 2.2. Inclusion and Exclusion Criteria

The inclusion criterion was a lifetime first episode of major depression. Occupational health care physicians and nurses were asked to recruit patients for the project. Participants were screened using the Finnish version of the BDI [10, 11] with a cutoff score of >9. For the current depressive episode, antidepressive drug use for less than six months and/or sick leave for less than one month was allowed. Subjects with schizophrenia group disorders, organic mental disorders, substance abuse disorders, or mental retardation were excluded. Moreover, subjects with depression that could not be treated in occupational health care services (psychotic symptoms or high suicide risk) or that required hospitalization were excluded. After being given a detailed description of the study, all participants provided written informed consent. The ethical committee of the Northern Ostrobothnia Hospital District, Oulu, Finland, approved the study in 2004.

### 2.3. Subjects

The participants were recruited from occupational health care units with about 120,840 clients (Figure 1). A total of 355 subjects were referred to the project, and 283 of them were randomized into the intervention (*N* = 142) and control groups (*N* = 141). Eight of the subjects were excluded at the baseline, so the number of participants was 275: 141 in the intervention group and 134 in the control group. After one year of followup, the intervention group consisted of 134 participants, 79.1% female, and the control group contained 100 participants, 92.0% female. 

### 2.4. Methods

The Structured Clinical Interview for DSM-IV (SCID I-II) [12, 13] was used as a diagnostic tool. The interview consists of two parts: SCID I, for axis I disorders, and SCID II, for personality disorders (PDs). SCID interviews were conducted by trained and experienced interviewers (mainly TR and SB). All cases were reviewed together with a senior researcher (KL), who has long experience of using the SCID. The severity of depression at baseline was defined in the SCID I interviews as mild, moderate or severe and using the Finnish version of the BDI [10, 11]. The range of the BDI score is 0–63: 0–9 indicating no depression, 10–16 mild depression, 17–29 moderate, and 30–63 severe depression. 

In the SCID II interviews, the presence of any comorbid PD was assessed. Obsessive compulsive personality disorder (OCPD) was used as a potential confounder as it has been found to be prevalent in the present sample [9]. Comorbid OCPD may cause greater functional impairment in patients than depressive disorder alone [14, 15].

Participants were asked to complete questionnaires, including basic socio-demographic information, details of their current work situation, and use of antidepressive medication. Marital status was dichotomized: married or cohabiting versus single. Basic education was categorized into three groups according to the duration of education: less than nine years, nine years (comprehensive school), and more than nine years. Vocational education was categorized into three groups according to the level and length of education: lowest or without any vocational education/polytechnic education/a degree from university or university of applied sciences. Social class was categorized into three groups and defined using a nine-level Finnish classification system based on the social appreciation of professions [16].

### 2.5. Early Eclectic Rehabilitative Intervention Program (EERIP)

The rehabilitation process was conducted by a multiprofessional working group consisting of a psychologist, social worker, psychiatrist, physician, and physiotherapist in a rehabilitation institute, the Oulu Deaconess Institute. The working group remained the same throughout the entire field study period of 2004–2009. The intervention consisted of two types of courses, of which the first and the second were research courses and the third and fourth were rehabilitation courses. The entire rehabilitation process took 6 months and included 31 active days. The research courses focused on individual vulnerability factors of depression, which varied from work-related and family-related stressors to person-related stressors. Based on individual stressors, each participant received tasks to be completed during the rehabilitation process. The research courses were arranged for groups including 3–5 participants. The courses consisted of two 5-day-long periods with 3-4 week intervals. During the intervals, participants focused on their individual tasks [8]. The rehabilitation courses were scheduled 3-4 months after the research courses. They consisted of one 14-day-long and one-7-day-long course with a 3-4-week interval and were performed in groups of 5–8 participants, not necessarily containing the same people as in the research courses. The group working methods were based on eclectic practice, including both cognitive behavioral and psychodynamic principles [8]. Participants were resident at the Oulu Deaconess Institute during the courses, that is, living away from their normal circumstances.

The aims of both the Research and Rehabilitation courses were to increase self-knowledge of depressive symptoms and to provide peer and social support. In the case of work-related stressors, collaboration with employers and occupational health care services was included in the process. This involved rehabilitation personnel visiting the participants' work places in order to identify potential changes in the working conditions to reduce work-related stress. In the case of subjects with family-related stressors, the family members or other close intimates were included in the process. Spouses were asked to participate in family therapeutic sessions when required. A psychophysical physiotherapeutic approach to depression was adopted, emphasizing the interaction between mind and body. The aim was that the depressed participants could, through physical and body training and the use of relaxation techniques, recognize the importance of body reactions [17, 18].

A comparison of differences in the management of depression, using either EERIP or conventional treatments that followed Finnish treatment guidelines [19], is described in Table 1.

### 2.6. Statistical Methods

The outcome of the participants during the one-year followup period was determined using the BDI. Differences in the BDI between the intervention and control groups, and any changes during the one year followup, were analyzed using four outcome measures based on the sum score of the BDI. Firstly, we recorded the proportion of participants whose BDI score was less than 10 points (i.e., no depression) at the end of one-year followup. Secondly, we examined the proportion of subjects whose BDI score had decreased more than 50% during the followup. Thirdly, the proportion of subjects whose BDI score had decreased by more than 9 points during the followup was calculated. Finally, the change in mean sum score of the BDI was analyzed. 

When appropriate, Chi square and Fischer's exact tests were used for bivariate comparison of categorical variables, and Student's *t*-test was used for continuous variables. A logistic regression model was used to examine the likelihood for the failure to decrease by 9 points or more in the BDI score during the one-year follow-up time. The covariates used in a logistic regression model were those which showed a statistically significant bivariate association with the study group. All tests were two-tailed, and a limit for statistical significance was set at *P* < 0.05. All statistical analyses were performed using PASW Statistic 18 [20].

We conducted an attrition analysis, comparing subjects who were included at the baseline, but who did not participate at the follow-up phase (*n* = 41), to those who did participate at the one-year follow-up phase (*n* = 234). Most of the dropouts (82.9%) were from the control group (*P* < 0.001). The drop outs did not, however, differ from the participants in terms of age, gender, OCPD, or the severity of depression measured by BDI or SCID I at the baseline.

We also used the last-observation carried forward (LOCF) approach, assuming that the BDI scores of the dropouts remained the same at followup as they were at baseline. In these analyzes, the number of subjects in the intervention group was 134 and in the control group 127.

## 3. Results

The sociodemographic and clinical characteristics of the subjects are shown in Table 2. The mean age for males was 44.6 years (standard deviation = SD 10.0) and 45.3 years for females (SD 8.1), (*P* = 0.639). Twenty-eight participants, 20.9%, in the intervention group and eight participants, 8.0%, in the control group were male. Of the participants, 169, 72.2%, were married or cohabiting and 112, 57.1%, were 40–50 years of age. Of the participants, 182, 77.8% were educated beyond the compulsory level. Of the participants, 137, 52.6%, had vocational education higher than the lowest level. Of the participants 139, 59.4%, had middle social status. The proportion of subjects with high social status was greater in the intervention group compared to the control group. Of the participants, 131, 56.7%, worked in the public sector. Of all sociodemographic variables, statistically significant differences between the intervention and control groups were found in gender (*P* = 0.007) and social class (*P* = 0.030). In the clinical variables, less than every third person used antidepressive medication at the start of followup. The prevalence of OCPD was almost twofold in the intervention group compared to the control group, and this difference between study groups was statistically significant (*P* = 0.028).

According to the SCID I interviews, 34.3% of participants in the intervention group had mild, 59.0% moderate, and 6.7% severe major depression at baseline. In the control group the respective rates were 49.0%, 45.0%, and 6.0% (*P* = 0.075). The mean BDI score at the beginning of the study was 20.8 (SD 7.3) in the intervention group and 19.3 (SD 7.4) (*P* = 0.136) in the control group and, after one year of followup, 9.1 (SD 9.1) and 8.8 (SD 8.1), (*P* = 0.858), respectively. The mean decrease in BDI scores in the intervention group was 11.6 (SD 10.0) and 10.8 (SD 9.8) in the control group. The decrease was statistically significant within both of the groups (*P* < 0.001).

Of the all four outcome measures, the only significant difference between the study groups was found in the decrease in BDI scores over 9 points during the one-year follow-up period. This was the case in two-thirds of the intervention group and in half of the control group (*P* = 0.013; Table 3). When this association was modeled with a logistic regression analysis (Table 4), after controlling for participants' gender, OCPD, and social class, the result remained statistically significant. When compared to the intervention group, the likelihood of control group members not having a decrease of 9 points or more in BDI score during the followup was 1.89 (CI 1.06–3.37, *P* = 0.030).

In the LOCF analyzes, three of the four outcome measures were significant. BDI scores decreased over 50% in 60.6% in the intervention group and 42.7% (*P* = 0.004) in the control group. BDI decreased over nine points in 60.6% in the intervention group and 39.5% (*P* < 0.001) in the control group. Mean decrease of the BDI scores was 10.9 (SD 10.0) in the intervention group and 8.0 (SD 9.7; *P* = 0.016) in the control group.

## 4. Discussion

The purpose of this study was to examine the effect of the EERIP on depressive symptoms in subjects with an acute onset of first ever depression. The main finding of this study was that, in first ever episodes of depression among working age employed people, the early eclectic rehabilitative intervention program (EERIP) may be more effective in reducing symptoms of depression than treatment as usual (TAU). However, the effect on symptom level of depression was only minimal. Out of four measures of symptom level changes, only one was statistically significant (a decrease in BDI score >9 points). Our findings support, in part, the hypothesis that the EERIP would be effective in reducing depressive symptoms. To the best of our knowledge, no similar earlier studies exist exploring the effectiveness of an eclectic rehabilitative intervention in working age persons experiencing first episode depression.

The results of rehabilitative interventions in early depression are contradictory. The positive influence of the interventions in managing depression has been observed in various studies [21–25]. A resource-building group intervention used to strengthen recovery from depression has been shown to improve mental health among employees with elevated levels of depression [26]. However, a systematic review by Furlan et al. [4] concluded that, to date, there is insufficient evidence to determine which interventions are effective in managing depression in the workplace. A recent Finnish cohort study of 50,000 employees conducted by Saltytchev (2012) did not find any evidence of the effectiveness of vocationally oriented medical rehabilitation amongst public sector employees [27]. The results in this study demonstrate that early eclectic rehabilitative intervention is not a particularly powerful tool, but it provides an additional useful option for the management of depression in employed people.

The EERIP included collaborative work with the participants' employers. The aim was to identify possible recommendations for changes in working conditions and environment in order to reduce work-related stress. The active collaborative work conducted during the intervention process may explain why the participants in the intervention group benefited from the EERIP. Andrea et al. [28] have encouraged the use of intervention studies to test whether changes in the work place or in the psychosocial work environment reduce depressive symptoms among employees. Dietrich et al. [29] have suggested that more tailored interventions, targeting depression directly, are needed in the workplace. There is a need for new strategies in clinical practice with regard to the psychosocial work environment and disability due to mental disorders [30, 31].

The EERIP provided the opportunity for the subjects to obtain peer support, to reduce the stigma associated with mental health, and to better understand the features of depression. Other workers and lay people may show ignorance with regard to the causes and treatment of mental disorders [32]. Peer support interventions versus usual care have been shown to be superior in reducing symptoms of depression [33]. In the present rehabilitative intervention, peer and social support were emphasized, with the focus on the role of social support via collaborative action with employers and family members. This may partly explain the better results in the EERIP group.

### 4.1. Limitations

This study had several limitations. Of the subjects, only 8% were male in the control group and 21% in the EERIP group, which limits our ability to generalize the results to males. The small number of males is probably due to characteristics of the population from which the sample was drawn. In many of the occupational health care units involved in the study, most clients were working in social and health care and education professions in which the majority of employees tend to be females. Moreover, a high number of the dropouts in the control group were male. One limitation may be that most of the dropouts were from the control group. It may be that the intervention motivated more subjects to participate than treatment as usual. The proportion of OCPD in our study was higher in the EERIP group than in the control group. In the EERIP group, there were more subjects belonging to the highest social group than the lower social groups. OCPD probably impairs recovery from depression [34]. However, we did not find any differences in the recovery between subjects with and without OCPD, or belonging to low or high/moderate social class. A followup period of one year may be too short to evaluate the long-term effect of the rehabilitative intervention. More time would be needed to fully implement cognitive tools and to establish new behaviors [25]. The EERIP took 6 months and included 31 active days. We had no detailed information of the resources and practices in the occupational health care units concerning the management of depression (i.e., treatment as usual), and we did not know to what extent the recommended guidelines for the treatment of depression were being followed [19]. The use of a self-report inquiry, like BDI, may be not as reliable as using rating scales or standardized psychiatric interview techniques in evaluating the severity of depression. However, the BDI is widely used in depression treatment studies [35].

### 4.2. Strengths

The strengths of this study include the use of a control group in a randomized design. Due to the inclusion and exclusion criteria, the subjects represent a group with first episode depression in working age people who did not have notable treatment for depression previously. The group was suffering from depression without other disorders, such as substance abuse or psychotic disorders. Diagnoses were made using an appropriate interview technique. The LOCF analyses supported our results with the full followup data. A notable factor in the intervention process was the multiprofessional working group and the fact that the group remained the same during the entire process (from 2004 to 2009), thus ensuring quality and consistency in the intervention process. To our knowledge, comparative studies focusing on this type of rehabilitative intervention among employed people have not been conducted previously.

## 5. Conclusions

The results show some potentially beneficial effect of EERIP intervention. The early eclectic intervention program may represent a useful addition to the management of the complex and multifactorial syndrome of depression, improving occupational care units' ability to help and treat employees presenting with a first ever episode of depression.

## Figures and Tables

**Table 1:** Recommendations in the early eclectic rehabilitative intervention program versus the finnish guidelines for managing depression: early eclectic rehabilitative intervention program versus the finnish guidelines.

Components	Intervention group (EERIP)^1^	Control group (FGL)^2^
Antidepressive medication	Yes	Yes
Cognitive or other individual therapy	Yes	Yes
Work orientation	Yes	Yes/No
Family orientation	Yes	Yes/No
Group therapy methods	Yes	No
Duration of intensive interventions	6 months	No recommendation

^1^EERIP: Early eclectic rehabilitative intervention group.

^2^FGL: Finnish guide lines for managing depression.

**Table 2:** Socio-demographic and clinical characteristics of the participants.

Variable	Intervention group *N* = 134		Control group *N* = 100		Difference
	*N*	%	*N*	%	*P*-value
Gender					
Male	28	20.9	8	8.0	0.007
Female	106	79.1	92	92.0	
Marital status					
Cohabiting	96	71.6	73	73.0	0.819
Other	38	28.4	27	27.0	
Age groups					
<40 years	33	24.6	21	21.0	0.781
40–50 years	62	46.3	50	50.0	
>50 years	39	29.1	29	29.0	
Basic education					
High	53	39.5	37	37.0	0.893
Medium	51	38.1	41	41.0	
Low	30	22.4	22	22.0	
Vocational education					
High	30	22.4	20	20.0	0.059
Medium	57	42.5	30	30.0	
Low	47	35.1	50	50.0	
Status of employer					
Public	74	56.1	57	57.5	0.635
Private	52	39.4	35	39.4	
Other	6	4.5	7	7.1	
Social status					
High	24	17.9	10	10.0	0.030
Medium	83	61.9	56	56.0	
Low	27	20.2	34	34.0	
Antidepressive medication					
Yes	38	28.4	29	29.3	0.885
No	96	71.6	70	70.7	
Obsessive-compulsive personality disorder					
Yes	41	30.6	18	18.0	0.028
No	93	69.4	82	82.0	

**Table:** (a)

BDI score changes	Intervention group (*n* = 134)		Control group (*n* = 100)		Difference
	*N*	%	N	%	*χ* ^2^-test, *P*-value
<10 at the end of the study					
No	51	38.6	39	39.4	0.907
Yes	81	61.4	60	60.6	
Decreased ≥50% during the study					0.329
No	46	35.7	39	42.4	
Yes	83	64.3	53	57.6	
Decreased >9 points during the study					0.013
No	46	35.7	17	46.7	
Yes	83	64.3	49	53.3	

**Table:** (b)

Mean changes in the BDI scores	Intervention group (*n* = 134)			Control group (*n* = 100)			*T*-test, *P*-value
	*N*	Mean	SD^1^	*N*	Mean	SD^1^	
	129	−11.6	10.0	92	−10.8	9.8	0.525

Note: ^1^SD: standard deviation.

**Table 4:** The effect of the intervention on changes in the BDI scores, using the logistic regression model.

Variable	Failure to have decrease in BDI scores > 9 (%^1^)	OR^2^	95% CI^3^	*P*-value
Group				
Control	46.7	1.89	1.06–3.37	0.030
Intervention (ref)	35.7			
Gender				
Male	50.0	1.79	0.85–3.77	0.104
Female (ref)	38.4			
OCPD				
Yes	47.4	1.49	0.79–2.81	0.172
No (ref)	37.8			
Social status				
Low	36.2	0.74	0.39–1.42	0.368
High + moder (ref)	41.7			

Note: 13 cases missing.

^1^Failure: BDI score did not decrease 10 points or more. The control group was compared with the intervention group taking into account gender, obsessive compulsive personality disorder (OCPD) and social group.

^2^OR: Odds Ratio.

^3^CI: Confidence Interval.
